# White matter hyperintensities and amyloid pathology in cognitively unimpaired aging: topographical, microstructural, and network-level evidence

**DOI:** 10.3389/fnagi.2026.2005174

**Published:** 2026-10-26

**Authors:** Sana Mohammadi, Minoo Sharbafshaaer, Maria Agnese Pirozzi, Francesca Trojsi, Sadegh Ghaderi, Yasser Iturria-Medina, Seyed-Mohammad Fereshtehnejad

**Affiliations:** 1 Department of Medical Sciences, School of Medicine, Iran University of Medical Sciences, Tehran, Iran; 2 School of Medicine, Hamadan University of Medical Sciences, Hamadan, Iran; 3 Department of Advanced Medical and Surgical Sciences, MRI Research Center (DAMSS), University of Campania “Luigi Vanvitelli”, Naples, Italy; 4 Neurology Unit, First Division of Neurology and Neurophysiopathology, AOU University of Campania “Luigi Vanvitelli”, Naples, Italy; 5 Department of Neurology and Neurosurgery, Montreal Neurological Institute, McGill University, Montreal, QC, Canada; 6 Montreal Neurological Institute, McConnell Brain Imaging Centre, McGill University, Montreal, QC, Canada; 7 Ludmer Centre for Neuroinformatics and Mental Health, Montreal, QC, Canada; 8 Pacific Parkinson’s Research Centre (PPRC), Djavad Mowafaghian Centre for Brain Health, University of British Columbia, Vancouver, BC, Canada; 9 Division of Neurology, Faculty of Medicine, University of British Columbia, Vancouver, BC, Canada; 10 Division of Clinical Geriatrics, Department of Neurobiology, Care Sciences and Society (NVS), Karolinska Institute, Stockholm, Sweden

**Keywords:** Alzheimer's disease, cognitively unimpaired older adults, diffusion MRI, white matter hyperintensity, β-amyloid

## Abstract

The frequent coexistence of white matter hyperintensities (WMHs) and β-amyloid (Aβ) pathology in cognitively unimpaired older adults complicates interpretation of vascular and neurodegenerative imaging findings. This narrative review synthesizes topographical, microstructural, and network-level evidence in cognitively unimpaired aging, distinguishing biomarker-characterized samples from supportive mixed or symptomatic cohorts. WMHs have heterogeneous etiologies. Posterior and some periventricular patterns are associated with cerebral Aβ in several cohorts, but lobar location and distance from the ventricles are overlapping classifications, and vascular associations are not confined to frontal or deep lesions. Independent and additive cognitive associations are more consistently reported than interactions. Longitudinal executive-function interactions reported in a dissertation and related abstract remain preliminary and non-independent. Some longitudinal studies associate baseline Aβ with subsequent WMH accrual, but findings are not uniform and do not establish causation. APOE-related findings vary with age, population, and amyloid burden. Diffusion MRI detects tissue abnormalities beyond visible lesions, although microstructural and disconnectome measures require prospective validation before they can improve individual prognosis. Anti-amyloid trial evidence identifies MRI safety considerations; effects on chronic WMH progression remain uncertain.

## 1 Introduction

Understanding the preclinical phase of dementia is critical for the development of effective strategies for early detection and intervention. Two major pathological processes converge during age-related brain aging: cerebrovascular disease and Alzheimer's disease (AD). Neuroimaging allows the visualization of the in vivo footprints of these processes, providing a critical window for the aging brain (Sperling et al., 2011).

White matter hyperintensity (WMH) is a neuroimaging marker visible as bright regions on T2-weighted fluid-attenuated inversion recovery (FLAIR) magnetic resonance imaging (MRI). It is primarily associated with cerebral small vessel disease (CSVD), advanced age, and vascular risk factors such as hypertension. Pathologically, WMH reflects underlying damage to the white matter (WM) of the brain, including demyelination and axonal loss (Tubi et al., 2020; Cha et al., 2024). Concurrently, preclinical AD pathology is characterized by the accumulation of β-amyloid (Aβ) plaques in the brains of cognitively unimpaired (CU) older adults and is considered one of the earliest events in the AD pathological cascade (Sperling et al., 2011). CU denotes clinical status; cognitively normal (CN) is retained only when describing terminology used by a source study. Preclinical AD requires biomarker evidence of AD pathology in a CU person; these terms are not interchangeable (Jack et al., 2024).

WMHs should be understood as a radiological phenotype with heterogeneous etiologies rather than as a single disease marker. In this review, vascular pathology refers primarily to cerebral small vessel disease (CSVD), hypertension-related injury, and other vascular risk-related mechanisms, whereas AD-type pathology refers to Aβ accumulation as measured by either positron emission tomography (PET) or cerebrospinal fluid (CSF) biomarkers. Although PET and CSF amyloid measures are related, they do not represent identical biological information, and their interpretation may differ depending on disease stage (Reimand et al., 2019). Clarifying these distinctions is essential for interpreting associations between WMHs, amyloid burden, and cognition in cognitively unimpaired older adults. These categories overlap: cerebral amyloid angiopathy (CAA) is an Aβ-related vascular process, and cortical amyloid biomarkers do not by themselves isolate vascular from parenchymal amyloid (Charidimou et al., 2017, 2022; Greenberg and Charidimou, 2018).

Thus, the core clinical challenge arises from the frequent coexistence of vascular and Alzheimer-type pathologies, which complicates early diagnosis and the design of disease-modifying clinical trials. Together, these two pathological processes may coexist, interact, or follow partially overlapping but non-identical trajectories in cognitively unimpaired older adults. However, the available literature does not support a single uniform model. Instead, the evidence suggests that WMHs should be viewed as a heterogeneous imaging phenotype whose meaning depends on lesion topography, underlying vascular burden, concurrent Aβ pathology, and white-matter microstructural integrity. The present narrative review, therefore, focuses on four linked questions: whether WMHs reflect primarily vascular or AD-related processes, whether amyloid burden predicts WMH progression or the reverse, whether diffusion-based microstructural abnormalities precede visible WMHs, and whether co-occurring pathology produces additive or synergistic cognitive effects. This structure is intended to reduce conceptual overlap and to distinguish etiologic mechanisms, imaging phenotypes, and clinical outcomes.

## 2 Methods

The initial narrative-review search was described as covering publications through October 2025 in PubMed, Scopus, Web of Science, and Embase. October 2025 denotes the publication cutoff, not a verified execution date; the exact initial search dates and database-specific exports are not available in the retained documentation. The strategy combined WMH terms (including “hyperinten*,” “FLAIR,” and “fluid-attenuated*”), amyloid terms (including “β-amyloid,” “amyloid-β,” “amyloid beta*,” and “Amyloid Protein A4”), and population terms (including “older adult*,” “cognitively normal,” “cognitively unimpaired,” “non-demented,” and “normal elderly”). English-language original cross-sectional or longitudinal studies relating an AD biomarker to WMH, diffusion, or network measures were considered. The synthesis distinguishes biomarker-characterized CU samples, broader CU samples without AD biomarker classification, and supportive mixed, symptomatic, inherited-AD, or other specialized cohorts. Only the biomarker-positive CU subgroup is described as preclinical AD; biomarker measurement alone does not make an entire CU sample preclinical AD. Pooled mixed-cohort results are not interpreted as CU-specific estimates.

A targeted presubmission check of primary publications and bibliographic records was conducted on 20 September 2026, rather than a repeat of the four-database search. A further targeted revision search on Oct 5, 2026 checked primary-study and PubMed records concerning the review's original themes, age/APOE effects, anti-amyloid safety, prevention trials, and WMH treatment outcomes. Later publications were incorporated when they addressed these questions; Strain et al. (2026), for example, was published online after the initial cutoff and is identified as supportive mixed-cohort evidence. Reviews supply background; a dissertation and related abstract are preliminary, non-independent reports. The available records do not permit reconstruction of historical deduplication, title/abstract screening, full-text exclusions, or screening responsibilities. Accordingly, no retrospective counts or claim of independent duplicate screening is made.

## 3 Results

### 3.1 The multifactorial etiology of white matter hyperintensities

This subsection addresses whether WMH topography can help distinguish vascular-associated from AD-associated white-matter injury. WMHs are a common neuroimaging finding in older adults and appear as hyperintense signals on T2-weighted FLAIR images. Figure 1 illustrates the progression from macrostructural, tissue, and lesion segmentation to an atlas-based estimate of the network-level disconnection associated with the segmented lesions. Although traditionally regarded as a surrogate marker of cerebral small vessel disease, studies in clinical small-vessel-disease cohorts also emphasize heterogeneity in WMH progression (Han et al., 2024). Standardized definitions distinguish the imaging phenotype from its possible pathological substrates (Duering et al., 2023).

**Figure 1:**
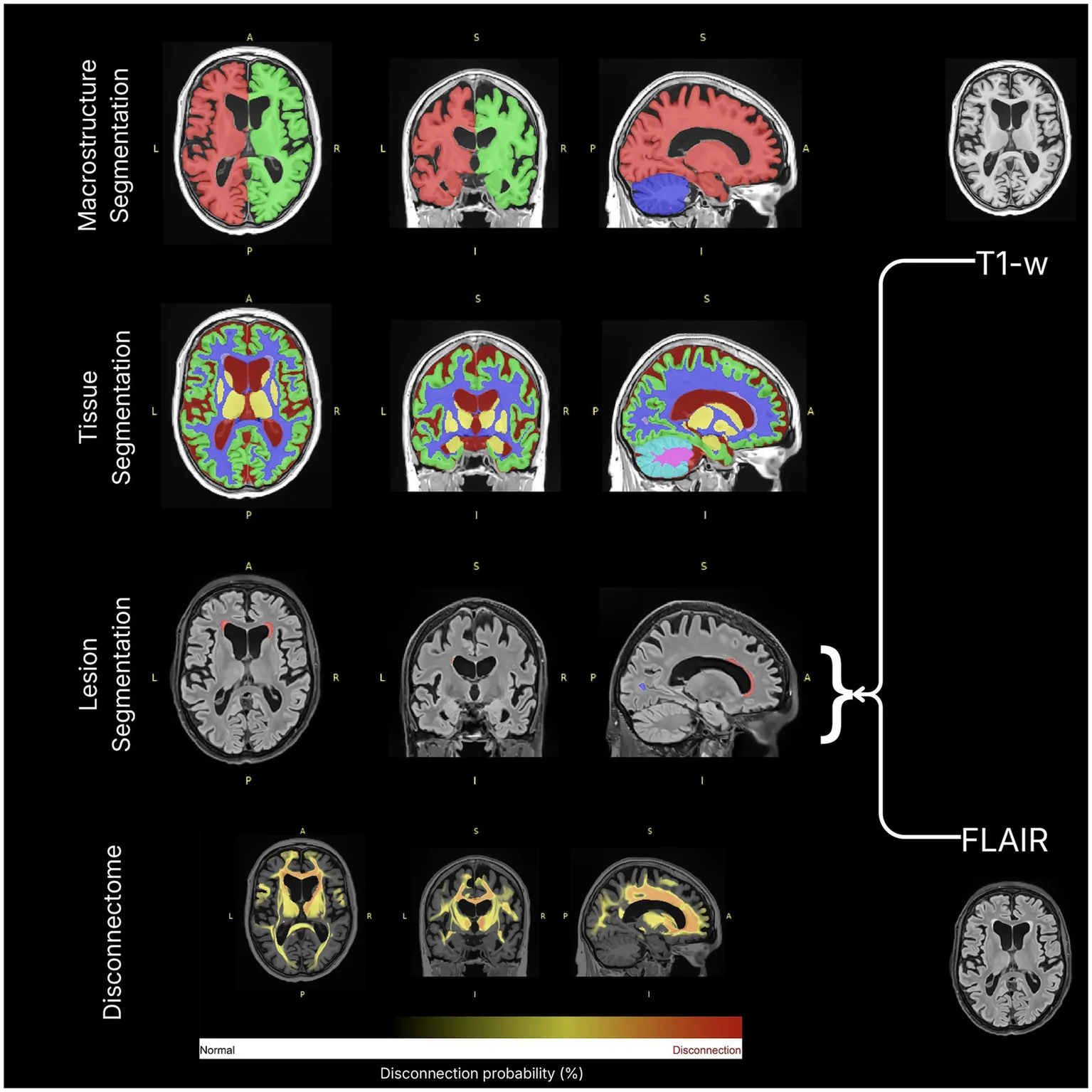
From structural MRI segmentation to lesion-based disconnectome estimation. An 82-year-old male ADNI participant with AD illustrates the processing pipeline. T1-weighted images support macrostructural/tissue segmentation; FLAIR supports WMH segmentation. The lesion mask is combined with normative tractography to estimate potential tract involvement, not to measure this participant's dMRI integrity. Warmer colors indicate greater atlas-derived model scores. “Normal” denotes little or no estimated involvement on this map, not verified normal tissue. “Disconnection probability (%)” denotes the software's model score, not a calibrated patient-specific likelihood of tract damage. This symptomatic participant is illustrative and does not represent the CU population. Processing used VolBrain (https://www.volbrain.net/) (Coupé et al., 2020; Denis de Senneville et al., 2020; Manjón et al., 2020; Kamraoui et al., 2022; Taghvaei et al., 2024).

For the illustrative WMH extraction example (Figure 2) 3 T 2D FLAIR scan in a woman with MCI was visualized. Brain extraction used SynthStrip (Hoopes et al., 2022), and preprocessing included N4 bias correction (Tustison et al., 2010), anisotropic diffusion denoising, and robust intensity normalization. Anatomical labels from robust SynthSeg (Billot et al., 2023) were mapped to the native FLAIR grid to define cerebral white-matter and reference regions. Three-class fuzzy c-means clustering (Bezdek et al., 1984) supported reference-intensity estimation; custom contamination-aware reference-tail calibration and local contrast defined lesion candidates and foreground/background markers. Marker-controlled watershed segmentation (Meyer and Beucher, 1990), with expansion capped at 2 mm beyond candidates, was followed by component filtering (minimum volume, 10 mm^3^). The saved native-space mask contained 7,105 voxels, corresponding to 26.24 mL. Images and masks were visualized without changing the segmentation. This example illustrates measurement steps and does not establish biomarker-defined preclinical AD or segmentation accuracy.

**Figure 2:**
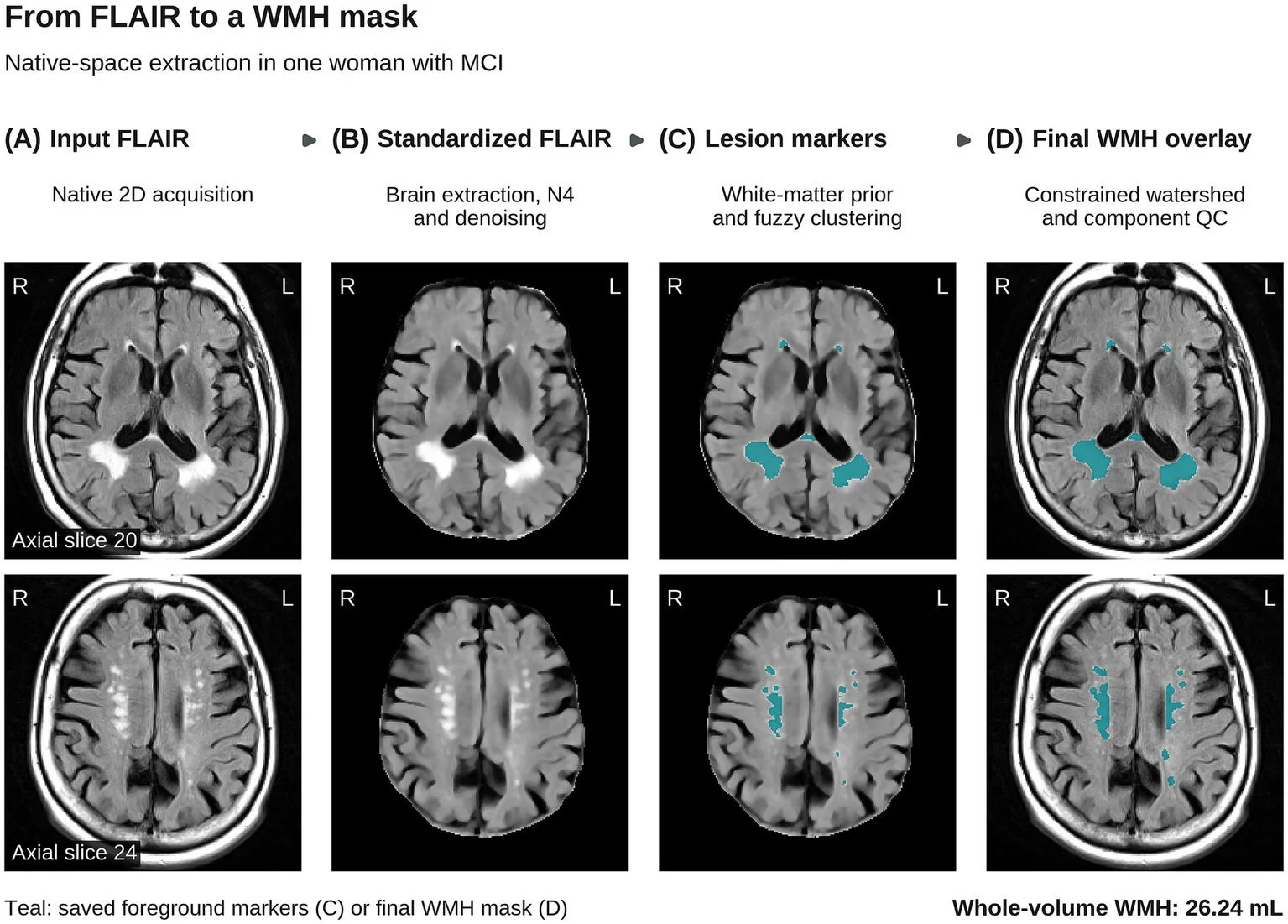
Native-space WMH extraction in a single illustrative FLAIR scan. Two matched axial levels from a 77.6-year-old woman with MCI show **(A)** the original 3-T 2D FLAIR image (from ADNI as well); **(B)** brain-extracted, bias-corrected, denoised, and robustly standardized FLAIR; **(C)** saved foreground markers over standardized FLAIR; and **(D)** the final WMH mask over the original FLAIR. Teal denotes foreground markers in **(C)** and the final binary mask in **(D)**, rather than a lesion probability. All panels share the same crop and native grid (0.86 mm × 0.86 mm × 5 mm); R and L indicate radiological orientation. Raw and standardized images use separate whole-brain display windows. The whole-volume WMH burden is 26.24 mL, rather than the burden in the two displayed slices.

WMHs are strongly associated with vascular risk factors, and hypertension has been consistently identified as a primary predictor. WMH burden is associated with post-stroke cognitive decline; pathological reviews also link WMHs to arteriolosclerosis, demyelination, axonal loss, and gliosis resulting from chronic ischemia and hypoperfusion (Ali H. F. et al., 2023; Inoue et al., 2023). Beyond CSVD, WMHs are also associated with AD-related processes that may overlap with vascular injury. Clinical and review evidence indicates that WMHs accompany AD and may have regional associations with amyloid that are not fully explained by measured vascular risk (Kim et al., 2020; Biesbroek et al., 2024). These findings indicate that distinct biological mechanisms may converge on similar neuroimaging phenotypes. Similarly, in a comprehensive adult lifespan study (30–89 years), Aβ+ status was associated with a stronger age-related increase in global WMH volume, and this association was found to be independent of hypertension (Caballero et al., 2020).

The anatomical location of WMHs provides critical insights into their etiology. Studies have increasingly differentiated patterns based on spatial distribution (Kim et al., 2020; Gu et al., 2025). Increased WMH volume in the superior longitudinal fasciculus (SLF) is specifically associated with an increased regional standardized uptake value ratio (SUVR) in the inferior parietal lobule (IPL) (Ali D. G. et al., 2023). Lesions adjacent to the lateral ventricles represent a pattern frequently associated with AD-related factors, including elevated Aβ burden and APOE ε4 carrier status (Brickman et al., 2009; Lee et al., 2016; Pålhaugen et al., 2021; Biesbroek et al., 2024). Deep WM lesions are also associated with hypertension and other vascular risk factors, but hypertension-related associations are not confined to deep WM (Debette and Markus, 2010; Hedden et al., 2012; Wardlaw et al., 2013; Gronewold et al., 2022; Phuah et al., 2022). A subcortical multispot WMH pattern has been associated with CAA, but is not diagnostic in isolation (Charidimou et al., 2017; Greenberg and Charidimou, 2018; Charidimou and Baron, 2025). Furthermore, amyloid load is correlated with a topographic pattern of WMH, predominantly involving periventricular WMH in the frontal and parietal lobes (Graff-Radford et al., 2019). Frontal WMHs have been associated with vascular risk and executive dysfunction, and posterior patterns with Aβ or memory in some studies, but neither association is anatomically or etiologically exclusive (Wu et al., 2002; Smith et al., 2011; Hedden et al., 2012; Biesbroek et al., 2017, 2024; Caballero et al., 2020; Garnier-Crussard et al., 2020; Chen et al., 2021; Pålhaugen et al., 2021; Phuah et al., 2022; Botz et al., 2023). Such topographical associations overlap across etiologies and do not establish a one-to-one mapping between lesion location and cognitive domain.

Lobar location and distance from the ventricles describe overlapping anatomical dimensions: a frontal lesion may be periventricular, deep, or juxtacortical. Frontal periventricular amyloid associations therefore do not contradict associations of deep frontal lesions with vascular risk. Differences in definitions, total-burden adjustment, vascular covariates, and possible CAA contributions further limit a binary interpretation. Table 1 compares representative measurement approaches.

**Table 1:** Representative WMH definitions and measurement methods.

Study	Sample and evidence context	Regional definition	Acquisition and quantification	Principal covariates
Marnane et al. (2016)	ADNI *n* = 698 (263 CU,435 MCI); pooled supportive; CU subgroup separable	PV frontal/parietal: maximal ventricular-horn extension (mm); occipital: ordinal visual score; no fixed-distance partition	Multicenter 3 T FLAIR; separate automated total WMH	Age, sex, APOE, vascular risk, pulse pressure, preventive medication, education, race/ethnicity
Pålhaugen et al. (2021)	DDI MRI *n* = 339; classification *n* = 303; mean age 63.7 ± 9.2 y; A+ SCD *n* = 24 is CU	Four ventricle-to-cortex layers × lobes; inner two PV, outer two deep; regional WM-volume normalization	Six scanners/five centers; 3D FLAIR/T1; Gaussian-mixture segmentation; scanner-specific acquisition	Age and scanner for amyloid comparisons; age-free Framingham score for vascular analyses
Phuah et al. (2022)	Supportive mixed ADNI *n* = 1,046; age 72.9 ± 7.6 y	Five data-driven patterns: juxtacortical, deep frontal, narrow PV, dorsal parietal, posterior/occipital; relative burden	Multicenter 3 T 2D FLAIR/T1; U-Net probabilities and spectral clustering	Age, sex, race, APOE, amyloid, vascular and CAA features; regional/total WMH
Biesbroek et al. (2024)	Supportive 11 memory-clinic cohorts *n* = 3,132; amyloid *n* = 1,273; age 71.5 ± 9 y	28 merged bilateral ICBM-DTI-81 atlas regions; tract/regional classification, not binary PV/deep	Site or central segmentations; T1 and FLAIR/T2; 1-mm MNI is resampling, not acquisition	Age, sex, study site; vascular score or Aβ; analyses with/without total WMH
Cha et al. (2024)	Supportive KBASE mixed *n* = 282; ages 55–90 y; 2-year follow-up	Global and lobar native-space ROIs; no PV/deep partition	Siemens 3 T mMR; sagittal 3D FLAIR; 1-mm slices; TR/TE 5000/173 ms; T1/FLAIR threshold algorithm	Age, sex, APOE ε4, vascular score, CDR-SB, baseline WMH for progression models

PV, periventricular; SCD, subjective cognitive decline with unimpaired objective cognition; DDI, Dementia Disease Initiation; CDR-SB, Clinical Dementia Rating Sum of Boxes; ROI, region of interest; TR/TE, repetition/echo time. Lobar location, distance from ventricle, and juxtacortical location overlap; study-specific categories are not equivalent. Acquisition is shown only where verified; heterogeneous protocols are not assigned invented field strengths or slice dimensions. ADNI-containing studies may share participants; exact overlap is not established.

Regional associations are informative at the group level but differ across parcellations and covariate models. A data-driven Alzheimer’s Disease Neuroimaging Initiative (ADNI) analysis associated amyloid with parietal and juxtacortical patterns, whereas its periventricular and posterior patterns were unrelated to the examined disease phenotypes (Phuah et al., 2022). Thus, lobar, distance-based, and tract-based categories are not interchangeable. Neither lesion location nor cortical amyloid alone establishes the cause of an individual WMH.

### 3.2 The interplay between WMHs and amyloid-β (Aβ) pathology

#### 3.2.1 Independent vs. interacting pathways

Evidence supports both independent and interacting relationships between vascular and amyloid-related processes, although these relationships appear to operate at different biological levels. In a cohort including CU participants and participants with mild cognitive impairment (MCI), linear regression analyses demonstrated significant independent effects of both hypertension (HTN) and CSF Aβ1–42 levels on WMH burden. Voxel-wise analysis indicated that 95% of HTN-related WMH voxels and 90% of Aβ-related WMH voxels were spatially distinct and non-overlapping (Al-Janabi et al., 2018). In contrast, Aβ burden and WMH volume interacted in their association with the myo-inositol-to-N-acetylaspartate ratio in the posterior cingulate and precuneus of cognitively unimpaired older adults, suggesting convergence on a downstream neurometabolic phenotype despite spatial separation of much of the lesion burden (Schreiner et al., 2018). The non-overlap percentages describe thresholded voxel-wise association maps, not the fraction of lesions with a proven vascular or amyloid etiology.

Collectively, these findings support partial independence rather than mutual exclusivity: vascular risk and Aβ pathology may contribute to white-matter injury through distinct spatial pathways, while their co-occurrence can still be associated with amplified downstream biological abnormalities.

#### 3.2.2 Temporal directionality and mechanistic hypotheses

Several longitudinal studies support an association between baseline Aβ pathology and subsequent WMH progression, although findings differ across cohorts. In CU individuals, greater amyloid burden, reflected by lower CSF Aβ1–42, was associated with greater WMH accrual over 2 years, independently of hypertension and age (Scott et al., 2016). In a mixed CU, MCI, and AD-dementia cohort, higher baseline Aβ burden was associated with greater subsequent WMH progression, with a stronger association observed in women (Cha et al., 2024). Aβ-positive status has also been associated with a stronger age-related increase in global WMH volume independently of hypertension (Caballero et al., 2020); however, because this age interaction does not itself establish temporal ordering, it should be interpreted as supportive rather than causal evidence.

Not all longitudinal evidence supports temporal coupling. One study found no temporal association between longitudinal cerebrovascular-disease and AD imaging biomarkers, suggesting that the two pathological processes may also evolve independently in some individuals (Cogswell et al., 2023).

Evidence supporting WMHs as antecedents of Aβ accumulation is less consistent. Baseline WMH burden predicted a subsequent increase in amyloid burden in a longitudinal study of patients with AD dementia (Grimmer et al., 2012). Regional studies have additionally shown that WMHs involving the superior longitudinal fasciculus are associated with greater Aβ deposition in a connected default-mode-network (DMN) node, the inferior parietal lobule (Ali D. G. et al., 2023), and that WMH topography correlates with Aβ deposition in early-accumulating cortical regions, including the medial orbitofrontal cortex, precuneus, and cuneus (Lorenzini et al., 2022). These spatial and connectivity-based observations are compatible with a hypothesized WMH-to-Aβ pathway but do not, by themselves, establish temporal causality.

Overall, some longitudinal cohorts support an Aβ-to-WMH temporal association, whereas others show little temporal coupling; evidence for the reverse pathway is limited and includes symptomatic populations. Nevertheless, independent and potentially bidirectional trajectories cannot be excluded, and the observational nature of most studies limits causal inference. A subsequent multicohort study associated higher baseline Aβ burden with faster regional WMH growth, including parietal regions, but included participants across clinical stages and remained observational (Strain et al., 2026). Table 2 distinguishes longitudinal findings from cross-sectional and pseudo-time inferences.

**Table 2:** Selected temporal and microstructural studies.

Study	Population and age	Design and follow-up	Principal measures	Finding and inference limit	Cohort reuse
Scott et al. (2016)	Biomarker-characterized CU; ADNI GO/2, *n* = 112; age 73.2 ± 6.6 y	24 months; *n* = 88 at final visit	CSF Aβ1–42; automated total FLAIR WMH	Baseline amyloid abnormality associated with later WMH accrual; temporal association is not causation.	ADNI; subset of earlier Scott report
Cha et al. (2024)	Supportive mixed CU/MCI/AD dementia; KBASE *n* = 282; ages 55–90 y	2 years	PiB-PET SUVR; automated global/lobar 3D-FLAIR WMH	Baseline Aβ associated with later WMH change; sex differences; pooled estimate is not CU-specific.	KBASE, distinct from ADNI
Cogswell et al. (2023)	Supportive mixed Mayo sample *n*=2,406 (1817 CU); age 74 ± 11 y	Trajectory models; 1–12 MRIs per participant	PiB-PET; WMH/TIV; genu FA	Little coupling of AD and vascular biomarker timing; modeling differs from short-interval change.	MCSA/ADRC; potential Mayo overlap
Liang et al. (2025)	Supportive mixed ADNI *n* = 532; CU A−154/A+107; CU ages 71.6 ± 6.6/72.6 ± 7.2 y	Pseudo-time; symptomatic validation interval 1.13 ± 0.54 y	Florbetapir/florbetaben PET; tract-specific FLAIR WMH and FA	Pseudo-time is not within-person chronology; longitudinal validation is symptomatic.	ADNI; possible shared participants
Benitez et al. (2022)	Biomarker-characterized CU *n* = 153 (A+51); eligibility 45–85 y	Baseline cross-sectional analysis	Florbetapir PET; DKI, tract-integrity modeling, FLAIR WMH	A+ group had greater diffusion restriction; reactive/biphasic sequence remains hypothetical.	MUSC community sample
Sun et al. (2025)	Supportive mixed ADNI *n* = 328 (174 CU), ages 55.3–93.9 y; GHABS *n* = 78	ADNI MRI *n* = 168; median 2.1 y (0.8–5.9); GHABS cross-sectional	Aβ-PET Centiloids; flortaucipir PET; total/lobar WMH; FA	Lower baseline FA associated with subsequent WMH increase; reverse relation nonsignificant.	ADNI possible overlap; GHABS separate
Strain et al. (2026)	Supportive mixed ADNI/AIBL/OASIS3 *n* = 1,243; age 71.7 ± 7.6 y	≥3 MRIs; duration not extracted from accessible abstract	PET Centiloids; TrUE-Net total/five regional WMH measures	Higher Aβ associated with regional WMH growth; observational medication findings are not treatment effects.	Multicohort; overlap possible; abstract-based extraction

CU denotes clinical status; biomarker-characterized CU samples contain A− and A+ participants unless stated otherwise. Only A+ CU subsets support biomarker-defined preclinical AD. Mean ± SD is shown where an observed range is unavailable; eligibility ranges are identified. Shared cohort names flag possible reuse, not verified participant-level overlap. Aβ, β-amyloid; ADNI, Alzheimer's Disease Neuroimaging Initiative; AIBL, Australian Imaging, Biomarkers and Lifestyle; DKI, diffusion kurtosis imaging; FA, fractional anisotropy; GHABS, Greater-Bay-Area Healthy Aging Brain Study; MCI, mild cognitive impairment; MRI, magnetic resonance imaging; OASIS3, Open Access Series of Imaging Studies 3; PET, positron emission tomography; SUVR, standardized uptake value ratio; TIV, total intracranial volume; WMH, white matter hyperintensity.

#### 3.2.3 Spatial co-localization and distribution patterns

The spatial distribution of WMHs is associated with regional cerebral Aβ burden, including posterior-predominant patterns (Botz et al., 2023). In a cohort including CU participants and participants with MCI, greater periventricular WMH burden in the parietal, occipital, and frontal regions was associated with elevated cerebral amyloid measured by both florbetapir PET and low CSF Aβ (Marnane et al., 2016).

A study reported that WMHs associated with low CSF Aβ1-42 levels were primarily located in the bilateral posterior corona radiata (Al-Janabi et al., 2018). Similarly, a recent systematic review also described that posterior periventricular WMHs coexisting with hippocampal atrophy are associated with early AD-related neurodegeneration (Gu et al., 2025).

A post-mortem study found that frontal white-matter lesions in AD are associated with both CSVD and AD-associated cortical pathology (McAleese et al., 2021). In addition, the WMH regions associated with amyloid pathology strongly correlate with lobar cerebral microbleeds (CMBs), consistent with a possible contribution of CAA to the relationship between amyloid and WMH in individuals without dementia (Graff-Radford et al., 2019). In a large cohort of CU older adults with preclinical AD, the baseline WMH volume was significantly greater in individuals with more than one microhemorrhage than in those with no microhemorrhage. In addition, longitudinal WMH accumulation was higher in participants with one or more microhemorrhages on the last MRI than in those without (Shirzadi et al., 2024). This finding supports the idea that an increased WMH volume may reflect early amyloid-related small vessel pathology.

#### 3.2.4 Biological modifiers of the Aβ–WMH association

Sex and genetic susceptibility may modify the association between Aβ pathology and WMH progression. A significant interaction between baseline Aβ deposition and sex has been reported, with higher baseline Aβ burden predicting subsequent WMH progression in women but not in men (Cha et al., 2024). APOE ε4 status may also influence this association. In individuals with MCI, APOE ε4 carriage was associated with greater periventricular WMH burden, but the association was no longer significant after accounting for concomitant Aβ burden (Bachmann et al., 2024). Conversely, another cohort showed no significant differences in sex, APOE genotype, global PiB SUVR, or Aβ-positive status between WMH-positive and WMH-negative groups (Wu et al., 2023). Thus, evidence for biological modification remains heterogeneous. Age may contribute to this heterogeneity. A cross-sectional UK Biobank study of 21,236 cognitively intact adults associated AD polygenic risk with tract microstructure more strongly in later life, largely reflecting APOE, but found no significant association with total WMH volume, including among participants aged ≥75 years (Chen et al., 2025). This sample was not selected for AD biomarkers and supplies supportive genetic evidence. Future longitudinal studies should prespecify age-by-APOE and sex interactions where adequately powered, without assuming a universal age threshold.

### 3.3 Microstructural alterations

Diffusion MRI (dMRI) provides complementary measures sensitive to white-matter microstructure beyond FLAIR-visible lesion burden. Recent research indicates that microstructural alterations indexed by reduced fractional anisotropy (FA) are strongly associated with WMHs and are associated with abnormal Aβ accumulation (Liang et al., 2025). White-matter alterations encompass both macrostructural burden (WMH volume) and microstructural integrity, particularly FA, in normal-appearing WM (NAWM) (Al-Janabi et al., 2018). In a community cohort without dementia, greater preceding cognitive decline was associated with later-life WMH burden, altered WM diffusion measures, and lower brain volumes (Orlando et al., 2022). Another recent study investigated the influence of WMH on hippocampal functional connectivity and gray-matter (GM) density in the medial temporal lobe of CU older adults. Perilesional injury and degeneration along affected tracts were discussed as possible explanations for abnormalities beyond visible WMHs; these mechanisms were not directly demonstrated by the functional-connectivity analysis. These processes are more commonly associated with vascular pathology, but may also interact with amyloid-related mechanisms (Wu et al., 2023).

Importantly, diffusion abnormalities may emerge before both clinical symptoms and overt macroscopic white-matter damage. In autosomal dominant AD, alterations in posterior parietal and medial prefrontal WM were detectable approximately 5–10 years before estimated symptom onset, indicating that microstructural white-matter alteration is an early component of the AD pathological cascade (Araque Caballero et al., 2018). This evidence concerns inherited AD and expected symptom timing; it does not establish the timing of visible-lesion formation in sporadic preclinical AD.

One study employed Aβ PET imaging ([^18^F]-florbetapir, FBP) to examine myelin integrity in WM. FBP retention in NAWM correlates with diffusion tensor imaging (DTI)-based measures of WM integrity, and has been proposed as a marker of myelin health (Moscoso et al., 2022). CSF Aβ1–42 levels were associated with alterations in NAWM-FA, whereas hypertension was only marginally associated with NAWM-FA alterations (Al-Janabi et al., 2018). In contrast, discrepancies observed in DTI metrics such as mean diffusivity (MD) and FA between diagnostic groups were largely attributed to unequal WMH load between the groups rather than alterations solely in NAWM, indicating the need for WMH adjustment in statistical comparisons (Svärd et al., 2017). Additionally, loss of WM integrity, quantified by an FA composite score, was associated with amyloid accumulation, GM atrophy, and CSF tau phosphorylation at T205 in autosomal dominant AD, indicating a specific association between tau phosphorylation at T205 and white-matter decline, independent of WMH volume and age (Strain et al., 2022). Moreover, serum clusterin levels were negatively associated with FA levels. Higher clusterin levels were primarily linked to the WM microstructure (FA) in several subcortical and thalamocortical (anterior internal capsule, posterior internal capsule, posterior thalamic radiation) and association (external capsule, superior longitudinal fasciculus) fiber pathways (Clark et al., 2021). However, serum clusterin was not significantly associated with WMH burden. WM retention of florbetapir remains an indirect and nonspecific correlate of myelin-related tissue properties, rather than a direct histological measurement.

Alongside the heterogeneous findings for Aβ burden and WMH progression, the relationship between tau pathology and macroscopic WMH burden is less consistent. Studies examining tau biomarkers and WMHs have reported heterogeneous findings, including null associations and region-dependent cortical tau differences in WMH-connected cortex (Soldan et al., 2020; Li et al., 2024; Zhang et al., 2024a). Similarly, no significant longitudinal relationship was observed in either direction between tau-PET deposition and WMH-volume change (Cha et al., 2024), and tau burden measured by PET was not associated with total WMH volume in cognitively unimpaired older adults (Graff-Radford et al., 2019). These findings do not establish the absence of a tau-related contribution; interpretation depends on the tau measure, clinical stage, follow-up, and statistical power.

By contrast, diffusion-based studies have reported associations between tau pathology and subtle white-matter injury. Across inherited-AD, clinicopathological, and mixed clinical cohorts, tau-related measures have been associated with altered FA or MD, although these diffusion measures do not uniquely identify axonal loss or demyelination (Kantarci et al., 2017; Strain et al., 2018, 2022). In former American football players, WMH burden was associated with higher CSF p-tau181 but not with CSF Aβ1–42, although the specific characteristics of this cohort limit generalizability to the broader cognitively unimpaired population (Ly et al., 2024). In cortical gray matter, a separate study reported that tau aggregation, but not amyloid burden, was strongly associated with increased cortical mean diffusivity, and mediation analyses indicated that tau-associated increases in cortical mean diffusivity partially mediated the relationship between Aβ burden and cognitive decline (Sun et al., 2024). The cortical result should not be treated as direct evidence for WM injury, and statistical mediation does not establish a causal pathway. Taken together, these studies support examining tau associations separately by tissue compartment and imaging measure.

Differences between macroscopic WMH and tau-related diffusion findings may partly reflect imaging scale and tissue compartment. Tau-associated axonal injury or secondary myelin changes may alter diffusion in NAWM without producing a FLAIR-visible lesion, whereas WMH volume is a nonspecific macroscopic endpoint with substantial vascular contributions (Svärd et al., 2017; Sun et al., 2025). This is a possible explanation, not an established tau-specific injury sequence. Diffusion indices are also nonspecific. Restricted early tau distribution, PET spatial resolution, and partial-volume effects can further weaken comparisons with global lesion burden (Zhao et al., 2019).

A study spanning normal cognition and MCI further reported anatomically distinct associations between the two pathologies and white-matter microstructure. WMH pathology was associated with reduced fractional anisotropy in projection and association fibers, whereas Aβ deposition was independently associated with reduced fractional anisotropy in the fornix and splenium of the corpus callosum (Chao et al., 2013). These findings indicate that WMHs and Aβ may affect partly different white-matter pathways even when both are associated with reduced microstructural integrity. Reported Aβ-by-WMH interactions were attenuated after adjustment for APOE ε4, limiting the evidence for independent synergistic effects.

Microstructural alterations in preclinical AD may not follow a simple monotonic pattern. In cognitively unimpaired older adults, Aβ-positive individuals showed greater diffusion restriction, characterized by higher fractional anisotropy and lower mean diffusivity, than Aβ-negative individuals (Benitez et al., 2022). Biophysical modeling suggested that this pattern may reflect an early reactive phase involving gliosis or myelin remodeling rather than established axonal degeneration. Thus, increased fractional anisotropy in early disease should not necessarily be interpreted as preserved white-matter integrity. The proposed reactive phase is a biological interpretation of cross-sectional differences, not an observed within-person sequence from repair to degeneration.

At the network level, lesion-based disconnectome mapping translates the spatial WMH mask into an estimate of the white-matter pathways likely to be interrupted. In virtual-lesion tractography, a FLAIR-derived WMH mask is projected onto normative diffusion tractography, and streamlines traversing the lesion are identified to generate tract-wise or voxel-wise estimates of disconnection (Figure 1). This approach complements lesion-volume measurement because individuals with similar WMH burdens may have different network consequences depending on the specific pathways affected. Importantly, the resulting disconnectome is an indirect atlas-based estimate rather than a direct patient-specific measurement of tract integrity and should be interpreted as model-based potential tract involvement, not a calibrated probability of actual tract damage in that individual (Taghvaei et al., 2024).

At the functional-network level, poorer white-matter microstructure within DMN tracts statistically mediated the associations of both age and AD pathology on DMN deactivation during a working-memory task (Brown et al., 2018). Reduced DMN deactivation was associated with greater AD pathology and lower fractional anisotropy in DMN WM, but not with total WMH burden. This finding suggests that disruption of DMN function in early AD may be related more closely to subtle tract-level microstructural damage than to the total volume of FLAIR-visible lesions.

Thus, diffusion findings indicate that visible WMHs represent one macroscopic component of a broader white-matter process. NAWM abnormalities and estimated disconnection provide complementary tissue and network information, but neither uniquely identifies histopathology. In a mixed ADNI clinical cohort, lower baseline FA in the superior fronto-occipital fasciculus was associated with faster subsequent WMH accumulation (standardized β = −0.564, 95% CI −0.714 to −0.413), whereas baseline WMH burden was not significantly associated with later FA change (Sun et al., 2025). These observations do not establish that every NAWM abnormality becomes a visible lesion.

### 3.4 Cognitive, functional, and clinical consequences of co-pathologies

The clinical relevance of coexisting WMH and AD pathology lies in whether they produce independent, additive, or interactive effects on cognition, everyday functioning, and clinical progression. Accordingly, the evidence in this section is organized by outcome rather than by etiological mechanism or temporal direction.

Independent associations refer to effects retained when both pathologies are modeled; additive effects are compatible with their sum on the model's outcome scale. An interactive effect requires a formal interaction test. Poorer performance in participants with both pathologies is insufficient by itself to establish interaction.

A substantial body of evidence supports independent and additive cognitive effects. In cognitively unimpaired older adults, a cerebrovascular imaging measure incorporating WMHs and infarcts and Aβ pathology independently predicted cognitive decline, while individuals positive for both pathologies showed greater decline consistent with an additive effect (Vemuri et al., 2015). Other studies found that the association between WMH burden and cognition remained significant after adjustment for Aβ pathology (Garnier-Crussard et al., 2020; Li et al., 2024). In a mixed CU/MCI cohort, region-specific analyses similarly showed that greater parietal and temporal WMH burden was associated with poorer executive function beyond the effects of regional Aβ deposition (Choo et al., 2025). These findings indicate that WM injury can contribute to cognitive decline independently of cerebral amyloid burden.

Preliminary reports describe interactive effects. A dissertation and a related conference abstract reported independent baseline associations and an Aβ-by-WMH interaction with longitudinal executive-function decline (Ali, 2023; Ali D. et al., 2023). A formal interaction between Aβ and a periventricular hyperintensity component was associated with poorer episodic memory in a pooled CU/MCI sample (β = −0.37, 95% CI −0.57 to −0.18); the basal-ganglia/thalamic component also included gray-matter hyperintensities (Bachmann et al., 2024). A subsequent longitudinal IDcog report found biomarker-by-WMH-by-time interactions in older adults without dementia, with CU sensitivity analyses, but its full sample included participants with CDR 0.5 and overlaps the same cohort rather than providing independent replication (Bachmann et al., 2026). In a mixed clinical cohort enriched for WMH burden, WMH–cognition associations were statistically mediated mainly by cortical thinning, with smaller serial pathways involving Aβ burden and cortical thinning (Ottoy et al., 2023). Although these mediation models are consistent with convergence between vascular injury, amyloidopathy, and neurodegeneration, their observational nature does not establish a causal sequence.

Network-level analyses help explain why total WMH volume alone incompletely predicts cognitive outcomes. In 481 cognitively intact ADNI participants, virtual-lesion tractography showed that estimated disconnection of the right inferior fronto-occipital fasciculus, frontal aslant tract, and superior longitudinal fasciculus statistically mediated the association between WMH volume and executive function. Estimated disconnection of the left uncinate fasciculus statistically mediated the association with memory, whereas right frontal-aslant-tract disconnection statistically mediated the association with language. Aβ positivity additionally moderated the relationship between WMH burden, tract disconnection, and executive-function performance (Taghvaei et al., 2024). These published results illustrate the relevance of disconnectome measures to domain-specific cognition.

Complementary diffusion studies also link microstructural white-matter integrity to cognitive and everyday functioning. Longitudinal loss of microstructural integrity in commissural and association tracts, including the genu and splenium of the corpus callosum and the superior longitudinal fasciculus, was associated with concurrent memory and gait decline (Tian et al., 2025). In Aβ-positive individuals with AD, greater posterior WMH burden, particularly in the splenium of the corpus callosum, was associated with poorer cognitive performance independently of Aβ burden (Garnier-Crussard et al., 2022). Higher fractional anisotropy in selected projection and association pathways was associated with greater independence in everyday health and safety activities (Clark et al., 2021), while macrostructural and microstructural properties of limbic and DMN pathways moderated the association between tau pathology and longitudinal memory decline (Qiu et al., 2024).

Evidence concerning clinical progression is more heterogeneous. Greater occipital WMH burden, together with Aβ pathology, predicted progression to dementia in a mixed CU/MCI cohort (Choo et al., 2025). In contrast, another non-demented cohort including MCI associated greater WMH volume with poorer cognitive performance and faster memory decline but not with future dementia conversion (Li et al., 2024). WMH burden has also been reported to increase the risk of conversion to MCI primarily among individuals with lower baseline CSF total-tau levels, without corresponding interactions with CSF Aβ1–42 or p-tau (Soldan et al., 2020). These findings suggest that the prognostic effect of WMHs may depend on lesion location, affected networks, and the accompanying biomarker profile.

Overall, the cognitive and clinical consequences of WMH and AD co-pathology are domain-specific rather than uniform. Executive function and processing speed appear particularly sensitive to frontal, parietal, and long-range tract disruption, whereas episodic-memory impairment is more associated with Aβ pathology but may be exacerbated by periventricular WMHs and disconnection of memory-related pathways. Both independent and interactive effects have been reported, although the observational evidence does not establish causality. Table 3 summarizes these outcomes and the limits of their interpretation.

**Table 3:** Selected cognitive and functional studies.

Study	Population and age	Follow-up	Principal measures	Finding and inference limit	Cohort reuse
Garnier-Crussard et al. (2020)	Biomarker-characterized CU lifespan IMAP *n* = 137, ages 19–85 y; older subgroup >60 *n* = 51	Cross-sectional	Florbetapir-PET SUVR; LST FLAIR total/lobar/callosal WMH	Executive associations after Aβ adjustment; formal WMH × Aβ cognitive interaction absent.	IMAP; other reports may reuse sample
Vemuri et al. (2015)	Biomarker-characterized CU MCSA *n* = 393, recruitment 70–89 y; A+126	Group medians 2.7–2.8 y; overall 1.0–7.7 y	PiB-PET; WMH/TIV and infarct vascular composite	Independent/additive global decline; amyloid×time β = −0.06 (SE 0.02), vascular×time −0.05 (0.02); no significant pathology interaction.	MCSA; potential Mayo reuse
Ali (2023); Ali D. et al. (2023)	Preliminary CU ADNI2 abstract *n* = 714; MMSE≥26; exact ages not extracted	Approximately 2 y	Regional/global Aβ-PET; FLAIR WMH; executive composite	Formal longitudinal executive interactions reported; dissertation and abstract are non-independent; no peer-reviewed full cognitive report.	Same project; ADNI reuse possible
Taghvaei et al. (2024)	CU ADNI *n* = 481, age 72.3 ± 6.3 y; amyloid status *n* = 421	Cross-sectional	PET/CSF Aβ; reviewed nnU-Net WMH/TIV; normative virtual tractography	Tract-disconnection associations and observational mediation; formal executive moderation; no individual prognostic validation.	ADNI; 50 normative HCP participants are reference data
Bachmann et al. (2024)	Supportive pooled IDcog *n* = 222 (171 CU, 51 MCI); ranges 50–89/53–89 y	Cross-sectional	Flutemetamol-PET Centiloids; LST LPA regional hyperintensity components	Periventricular×Aβ episodic-memory β = −0.37 (95% CI −0.57 to −0.18); no multiplicity correction; BGT includes GM lesions.	IDcog; later longitudinal report reuses cohort
Choo et al. (2025)	ADNI 200 CU and 523 MCI; CU estimates separable; age not extracted	Cross-sectional cognition; progression follow-up not extracted	Regional/lobar amyloid and WMH; assay/algorithm not extracted	CU temporoparietal-WMH/executive association; progression results include MCI.	ADNI; primary abstract extraction only
Tian et al. (2025)	Supportive BLSA adults ≥60 y; *n* = 775 overall; DTI307; WMH331 in methods	WMH 5.3 ± 2.5 y; DTI 5.2 ± 2.5 y	FLAIR/T1 WMH; tract FA/MD; plasma Aβ42/40 and tau	Concurrent memory/gait decline and tract deterioration; not a formal WMH × Aβ interaction or A+-defined CU prognosis.	BLSA; overlapping modality subsets

Representative studies illustrate clinical and biomarker distinctions; the table is not an exhaustive study count. Primary abstracts are identified where full-text metadata could not be verified. Ali's dissertation and related abstract are one preliminary evidence source. The Tian report gives WMH *n* = 331 in Methods and *n* = 339 in its abstract; the Methods count is shown. AD, Alzheimer's disease; BGT, basal ganglia/thalamus; BLSA, Baltimore Longitudinal Study of Aging; CSF, cerebrospinal fluid; CU, cognitively unimpaired; DTI, diffusion tensor imaging; GM, gray matter; HCP, Human Connectome Project; IMAP, Imagerie Multimodale de la Maladie d'Alzheimer à un stade Précoce; MD, mean diffusivity; MMSE, Mini-Mental State Examination; MCSA, Mayo Clinic Study of Aging; SE, standard error. Other abbreviations are defined in Table 2.

### 3.5 Anti-amyloid treatment and trial relevance

#### 3.5.1 Antibodies, ARIA, and chronic WMH

Aducanumab, lecanemab, and donanemab reduce cerebral Aβ biomarkers in early symptomatic AD; their pivotal studies provide supportive treatment and safety evidence rather than direct preclinical evidence (Budd Haeberlein et al., 2022; Sims et al., 2023; van Dyck et al., 2023). Amyloid-related imaging abnormalities with edema (ARIA-E) comprise vasogenic edema or sulcal effusion, including potentially reversible FLAIR changes. ARIA with hemorrhagic features (ARIA-H) comprises microhemorrhages or superficial siderosis on susceptibility-sensitive imaging; neither category is equivalent to chronic WMH (Salloway et al., 2022; Zimmer et al., 2025). APOE ε4 homozygosity and pretreatment hemorrhagic CAA markers increase ARIA risk. Descriptive Clarity AD analyses reported ARIA-E frequencies of 11.3%, 12.2%, and 19.3% across baseline age-related white-matter-change categories of absent lesions, focal lesions, and beginning confluence, respectively (van Dyck et al., 2025). These categories are not Fazekas grades, and these observations do not establish an independent WMH effect.

Direct treatment evidence for chronic WMH remains limited. An A4 secondary analysis found no detectable solanezumab effect on WMH growth in 1,129 participants (*p* = 0.50); an exploratory treatment-by-WMH-growth interaction for cognitive change involved a postrandomization measure and does not validate baseline treatment selection (Lin et al., 2025). Solanezumab targets soluble monomeric Aβ, so this result cannot be extrapolated to plaque-clearing antibodies. A nonrandomized study of 20 lecanemab-treated early symptomatic patients and 110 matched untreated patients reported WMH reductions in 80% of treated patients after a median of 13 administrations (Shi et al., 2026). This small, abstract-accessible observational report cannot establish causal lesion reduction or benefit in CU participants. Longitudinal analyses must distinguish chronic WMH from ARIA-E-related FLAIR changes.

#### 3.5.2 WMH in prevention trials

WMH currently has a clearer role in eligibility and MRI safety assessment than as an established enrichment biomarker or efficacy endpoint. AHEAD 3–45 uses amyloid PET to assign CU participants to intermediate- or elevated-amyloid trials, with amyloid PET and the Preclinical Alzheimer Cognitive Composite 5 (PACC5) as respective primary outcomes; APOE ε4 and geographic region stratify randomization (Rafii et al., 2023). TRAILBLAZER-ALZ 3 uses plasma p-tau217 for pathology eligibility and a clinical-progression primary endpoint in the baseline Clinical Dementia Rating (CDR)-Global = 0 population; its enrollment also includes CDR 0.5 participants (Yaari et al., 2025). Severe white-matter disease and substantial hemorrhagic abnormalities are exclusion considerations. AHEAD excludes > 4 microhemorrhages or any superficial siderosis; TRAILBLAZER-ALZ 3 excludes > 4 microhemorrhages or > 1 area of superficial siderosis. These programs do not use WMH as a primary endpoint.

Lecanemab and donanemab appropriate-use recommendations operationalize severe WMH as Fazekas grade 3; this is a practice-guidance threshold rather than a uniform volumetric cutoff across trials (Cummings et al., 2023; Rabinovici et al., 2025). Exploratory WMH analyses should prespecify outcomes, harmonize longitudinal segmentation and FLAIR acquisition, record scanner changes, and centrally adjudicate ARIA. Visual grading and volumes answer different questions, and WMH change alone cannot establish a treatment effect. Vascular-predominant versus amyloid-predominant profiles require joint assessment of vascular risks, WMH distribution, lacunes, lobar microbleeds, cortical superficial siderosis, enlarged perivascular spaces, APOE, and AD fluid/PET biomarkers. No single feature assigns lesion etiology.

## 4 Discussion

The reviewed evidence does not support a single linear model linking WMHs and Aβ pathology. Instead, it points to a layered framework in which vascular risk, Aβ burden, lesion topography, microstructural white-matter injury, and cognitive phenotype interact at different stages of the AD continuum. From this perspective, WMHs should not be interpreted simply as a vascular marker or an AD marker, but as a heterogeneous imaging phenotype whose biological meaning depends on regional distribution and the presence of concurrent neurodegenerative processes.

Observational temporal associations cannot determine which intervention prevents cognitive decline. Core biomarker-characterized CU evidence includes the association between baseline CSF amyloid abnormality and two-year WMH accrual reported in a longitudinal study (Scott et al., 2016). Supportive mixed-cohort studies also associate baseline Aβ with later WMH progression (Cha et al., 2024; Strain et al., 2026), whereas other trajectory analyses show little temporal coupling (Cogswell et al., 2023). Symptomatic two-visit validation and amyloid-ordered pseudo-time analyses provide additional hypotheses rather than CU-specific chronology or causal effects (Liang et al., 2025). This heterogeneity argues against a universal Aβ-to-WMH sequence.

An alternative hypothesis (Ali D. G. et al., 2023) proposes that primary damage to the WM from underlying vascular disease may precede and be associated with subsequent Aβ accumulation in connected cortical regions. In this view, SVD is hypothesized to act upstream of some AD-related changes. Cross-sectional observations are compatible with this model, showing that WMH volume in a specific WM tract, the SLF, was associated with increased regional Aβ deposition in an anatomically connected cortical area, the inferior parietal lobule. This finding supports a “retrograde hypothesis,” in which distal axonal injury related to small vessel disease (manifesting as WMHs) may promote increased Aβ burden in anatomically connected neuronal populations, thereby contributing to AD pathogenesis. The same cross-sectional pattern is also compatible with shared vulnerability or reverse effects; distinguishing these explanations requires additional temporal and mechanistic evidence.

A third perspective suggests that the two pathologies may not have a direct causal relationship but rather function as largely independent and additive contributors to cognitive decline. This view is supported by evidence modeling both processes as separate predictors of cognitive outcomes. Based on this hypothesis, vascular and amyloid pathologies were independent predictors of cognitive decline in CU older adults, with effects that were additive rather than synergistic (Vemuri et al., 2015). Similarly, a dissertation and related abstract reported independent baseline associations (Ali, 2023; Ali D. et al., 2023). These two reports are preliminary and non-independent. The same study also noted a statistically significant interaction on longitudinal changes in executive function, suggesting a hypothesis for follow-up rather than demonstrating a changing biological interaction across disease stages.

This unresolved debate between causal pathways motivates a shift in focus, from visible WMH lesions to the subtle, antecedent microstructural changes in WM integrity that may hold the key to the earliest interactions. This ongoing debate motivates testing whether interventions in preclinical AD benefit from stratification, targeting vascular health in some individuals, amyloid pathways in others, or both simultaneously (Zhang et al., 2024b). Based on the evidence reviewed, we present an integrative framework (Figure 3), which distinguishes reported associations among vascular factors, Aβ, WMHs, microstructure, networks, and cognition from unresolved causal hypotheses.

**Figure 3:**
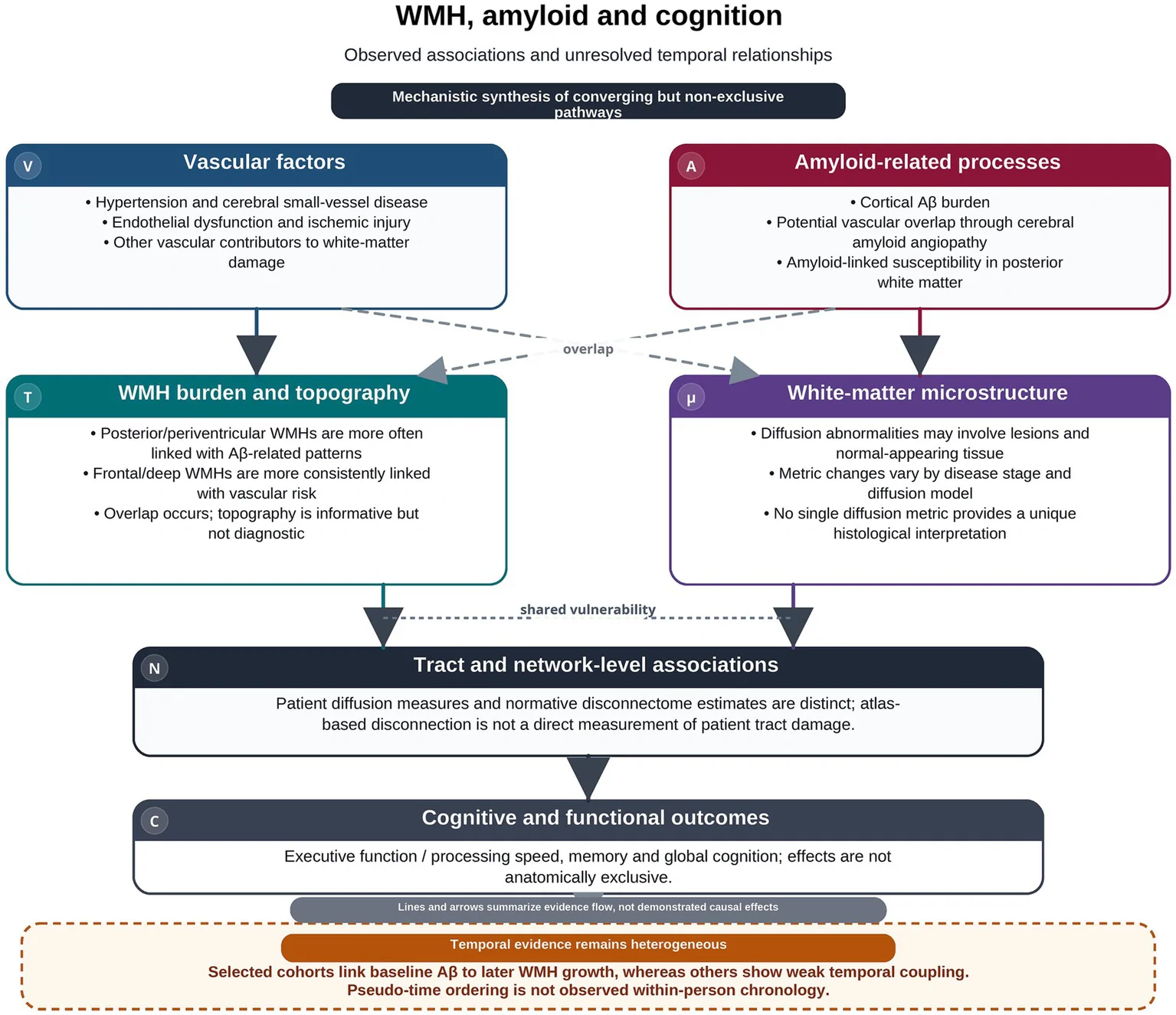
Evidence-informed framework linking vascular factors, amyloid pathology, WMHs, microstructure, networks, and cognition. Solid connectors summarize reported associations, not causal pathways or mutually exclusive lesion etiologies. Regional patterns overlap, and statistical interactions vary by cohort and outcome. The separate temporal-evidence inset distinguishes longitudinal associations from unresolved causal hypotheses; it does not specify a universal disease sequence. Diffusion measures and normative lesion-based disconnection estimates are indirect tissue and network measures, respectively. Executive function, processing speed, memory, and global cognition are not uniquely assigned to individual lesion locations. Aβ, β-amyloid; CAA, cerebral amyloid angiopathy; CSVD, cerebral small vessel disease; FA, fractional anisotropy; MD, mean diffusivity; NAWM, normal-appearing white matter; WMH, white matter hyperintensity. Lobar and distance-based WMH categories in the illustration overlap and are not universal etiologic classes.

The main value of the current literature lies not in proving a single direction of causality, but in showing that the same radiological phenotype can emerge from partially different biological pathways. This is why studies based only on global WMH volume are often difficult to interpret: they may merge vascular, amyloid-related, and mixed lesions into one outcome. Future work should therefore preserve lesion topography, distinguish PET- and CSF-based amyloid measures, and test whether regional WMHs and microstructural injury predict different downstream outcomes.

Visible WMHs may be preceded by subtler diffusion abnormalities, but lesion trajectories are heterogeneous. The increased diffusion restriction observed cross-sectionally in Aβ-positive CU participants has motivated a possible reactive phase involving gliosis or myelin remodeling (Benitez et al., 2022). A subsequent degenerative phase is hypothesized rather than demonstrated by that study. A single diffusion measure therefore cannot establish a linear or biphasic injury mechanism. Figure 4 summarizes the complementary information provided by FLAIR and dMRI.

**Figure 4:**
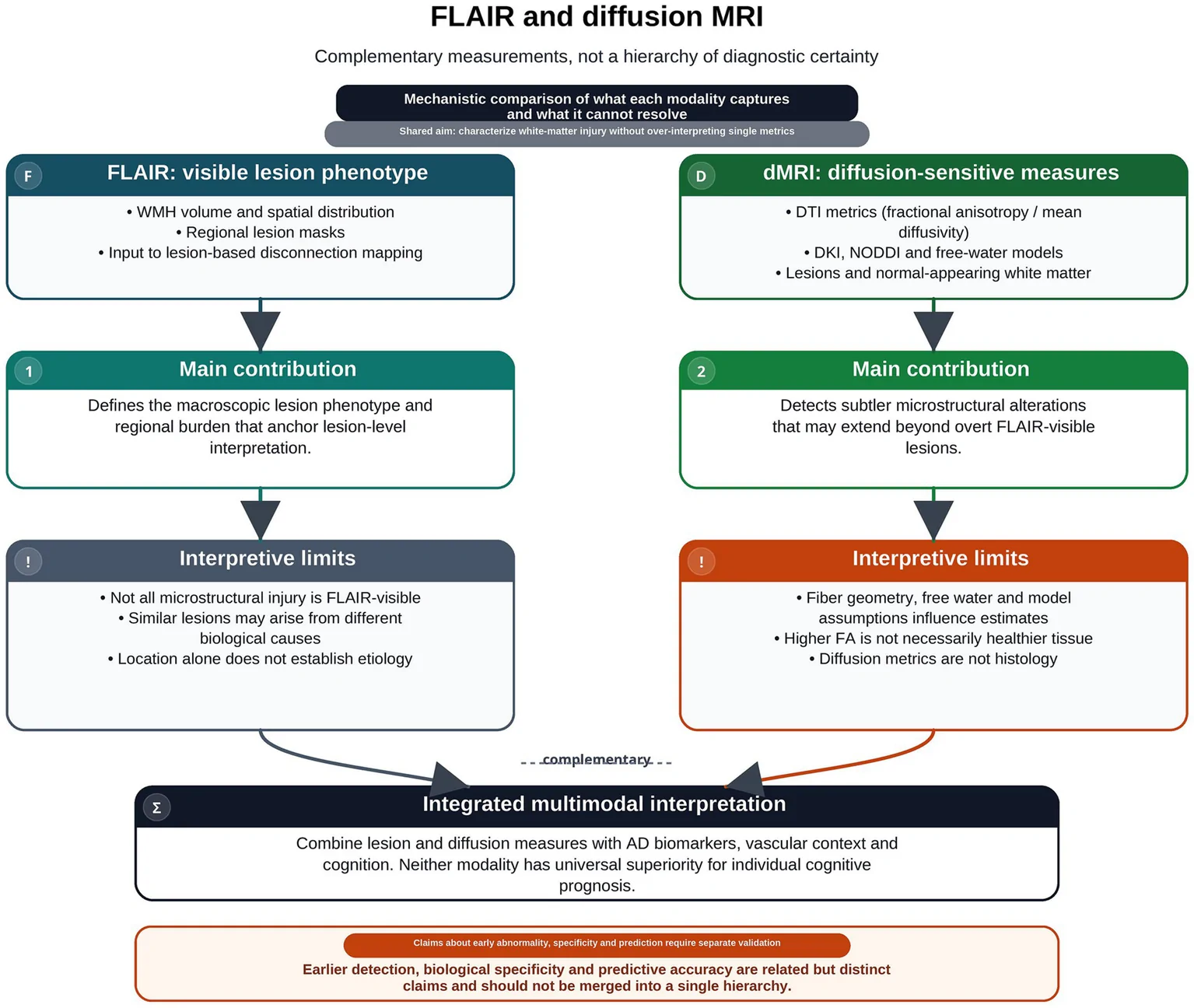
Complementary information from FLAIR and diffusion MRI. FLAIR characterizes visible WMH burden and topography, whereas diffusion MRI (dMRI) samples microstructural properties in lesions and normal-appearing white matter. Neither modality uniquely identifies the underlying histopathology. Diffusion indices are influenced by fiber geometry, extracellular water, acquisition, and model assumptions; higher FA does not necessarily indicate healthier tissue. Combining lesion location, microstructure, network estimates, AD biomarkers, vascular context, and cognition may improve characterization, but comparative prognostic superiority requires direct validation. The figure is a measurement framework, not a demonstrated temporal or causal sequence. AD, Alzheimer’s disease; DKI, diffusion kurtosis imaging; DTI, diffusion tensor imaging; FA, fractional anisotropy; FLAIR, fluid-attenuated inversion recovery; FW, free water; MD, mean diffusivity; NAWM, normal-appearing white matter; NODDI, neurite orientation dispersion and density imaging; WMH, white matter hyperintensity.

The impact of these microstructural changes is particularly evident within large-scale brain networks vulnerable to AD pathology. Research posits that disruptions in the function of the DMN are more strongly associated with alterations in WM microstructure (specifically, lower FA) rather than with the total volume of visible WMH (Brown et al., 2018). Reinforcing this point, the study observed that the core WM pathways of the DMN were spared from WMH, suggesting that diffusion measures can provide information not captured by total WMH burden in that sample. Complementary PET evidence suggests myelin-related tissue differences outside visible WMHs (Moscoso et al., 2022). They showed that the signal from [^18^F]-florbetapir PET in NAWM is associated with DTI measures and is predictive of future clinical decline. Collectively, these findings are compatible with microstructural abnormalities preceding some visible lesions, but do not establish a universal sequence leading from altered diffusion to cognitive deficits and WMHs. Clinically, these findings suggest that DTI and other microstructural imaging techniques may detect abnormalities before FLAIR-visible lesions in selected cohorts; superiority for individual prognosis requires direct validation (Rieckmann et al., 2016; Sun et al., 2025).

Added prognostic value should be tested prospectively by comparing a prespecified model containing age, vascular risk, amyloid status, and total WMH burden with the same model plus regional WMH, diffusion, or disconnectome measures. Outcomes should include meaningful cognitive decline, loss of everyday function, or incident MCI/dementia. External validation should assess calibration, discrimination, and decision usefulness where a clinical decision is intended (Collins et al., 2024). Better biological characterization alone does not demonstrate better individual prognosis.

Understanding which specific cognitive domains are affected during the preclinical stage is crucial for improving early detection, tracking disease progression, and designing sensitive endpoints for clinical trials. The evidence suggests that the interplay between WMH and Aβ pathology does not affect all cognitive functions equally, with some domains showing particular vulnerability. Several reports link the combination of WMH and Aβ pathology to declines in executive functions, which encompass higher-order cognitive processes such as planning, problem-solving, and cognitive flexibility (Garnier-Crussard et al., 2020; Ali, 2023; Ali D. et al., 2023; Taghvaei et al., 2024). This pattern supports assessing non-amnestic outcomes alongside memory. Alongside the findings regarding executive function, the data on episodic memory, the ability to recall personal experiences, are more variable in the preclinical stage. Preliminary analyses in clinically normal individuals described executive-function associations; the dissertation also examined memory, whereas the related abstract focused on executive function (Ali, 2023; Ali D. et al., 2023). While some evidence does connect WM damage to memory deficits, it was found that WMH-related disconnection in the left uncinate fasciculus was associated with memory performance (Taghvaei et al., 2024). These studies do not provide a uniform head-to-head comparison establishing stronger effects on executive function than on memory.

Together, these pathologies are associated with cognitive trajectories across the studied populations. A dissertation analysis suggests that WMH-dependent changes in baseline cognitive performance are related to both a direct effect of the WMH burden and an indirect effect that is statistically mediated through Aβ burden (Ali, 2023). In a separate cohort including participants with MCI, higher baseline WMH volume was associated with a more rapid decline in memory over time. However, baseline WMH volume did not predict conversion to dementia diagnosis, highlighting the complex relationship between structural brain changes and clinical outcomes. This observation challenges the assumption that structural lesion burden directly translates into clinical progression (Li et al., 2024). In summary, the findings support evaluating executive function and processing speed alongside memory, rather than establishing that one domain should replace another as a clinical-trial endpoint.

The functional consequences of both WMH and Aβ pathology are strongly influenced by their spatial distribution within the brain and their impact on critical brain networks. Therefore, a topographical approach that considers where these pathologies occur is strategically important to understand their clinical effects. The DMN plays a central role in the pathophysiology of AD (Greicius et al., 2004; Botz et al., 2023). Key nodes of the DMN, particularly the precuneus, parietal cortex, and posterior cingulate cortices, are discussed in regional iron and perfusion reviews as well as amyloid-staging studies (Cho et al., 2016; Grothe et al., 2017; Palmqvist et al., 2017; Mattsson et al., 2019; Sakr et al., 2019; Pfeil et al., 2021; Mohammadi et al., 2024; Ghaderi et al., 2025). The amyloid-staging studies identify early Aβ accumulation in overlapping DMN regions. The integrity of the DMN is dependent on the health of long-range WM tracts that connect its cortical hubs. Damage to these tracts may be associated with increased vulnerability to pathology. The presence of WMH in the SLF was associated with higher Aβ deposition in a connected DMN node, the IPL (Ali D. G. et al., 2023). This suggests that vascular-related WM damage in specific tracts may disrupt connectivity and be associated with vulnerability to AD pathology.

Furthermore, not all WMH are equivalent in their association with AD pathology. Research has begun to identify specific topographical patterns of WMH that are more strongly linked to the presence of cerebral amyloid. This topographical convergence suggests that the location of WMH, particularly in posterior periventricular regions (Marnane et al., 2016; Graff-Radford et al., 2019; Taghvaei et al., 2024), may motivate biomarker-informed investigation of amyloid-related processes, but cannot distinguish them from age-related or other vascular injury in an individual.

The APOE ε4 allele is a well-established risk factor for both late-onset AD and CAA, primarily by influencing Aβ clearance and promoting its aggregation (Yamazaki et al., 2019). However, its role extends beyond amyloidopathy; it has also been independently linked to cerebrovascular dysfunction, including blood-brain barrier breakdown and altered lipid metabolism, which may contribute to white-matter injury. In participants with MCI, the association between APOE ε4 and periventricular WMH was no longer significant after accounting for Aβ burden (Bachmann et al., 2024). This attenuation is compatible with shared variation with amyloid burden, but does not demonstrate mediation or exclude vascular contributions. APOE ε4 dosage has also been associated with altered WM microstructure in CU middle-aged adults (Operto et al., 2018). These observations justify evaluating APOE-related heterogeneity, but do not by themselves establish an accelerated WMH trajectory or identify a subgroup with proven benefit from combined treatment. Age-stratified analyses and continuous age-by-APOE models may help distinguish genetic associations with microstructure from those with visible lesion burden, without treating age 75 as a universal threshold (Chen et al., 2025).

The interpretation of the current evidence should account for several pervasive methodological limitations across the field. Many studies investigating this relationship have a cross-sectional design, which can identify associations but cannot establish causality or temporal ordering. Longer longitudinal follow-up is required to fully characterize the slow-progressing interplay between WMH and AD pathologies. Moreover, major research cohorts, such as the Alzheimer's Disease Neuroimaging Initiative (ADNI), often include exclusion criteria that limit the participation of individuals with significant cerebrovascular disease. This selection limits generalizability and may change the magnitude of WMH associations and interactions; the direction of bias cannot be assumed. Furthermore, the term “white matter hyperintensity” is a descriptive radiological term, not a specific pathological diagnosis. WMHs reflect heterogeneous underlying pathological processes, including SVD related to hypertension as well as AD-specific mechanisms. This heterogeneity confounds the interpretation of findings, as the cause of the WMH is not always clear from the imaging alone (Stewart et al., 2021; Solé-Guardia et al., 2023; Gu et al., 2025), and many studies do not establish the etiology of individual lesions.

WMH quantification ranges from ordinal visual ratings, such as Fazekas scores, to automated volumetric segmentation. Visual ratings summarize periventricular and deep lesion appearance; volumes measure lesion extent but depend on training data, intensity thresholds, registration, and quality control. Field strength, 2D versus 3D FLAIR, slice thickness, and partial-volume effects affect lesion detection and regional allocation. Global volumes can obscure regional patterns, whereas different regional definitions can create apparently inconsistent associations. Adjustment for total WMH or intracranial volume addresses different questions from adjustment for vascular risk, APOE, or CAA markers; total-burden adjustment can alter apparent regional amyloid associations (Duering et al., 2023; Biesbroek et al., 2024).

To resolve these limitations, future investigations must prioritize longitudinal, multimodal designs and harmonized WMH segmentation frameworks. Crucially, future study protocols should prespecify the interpretation of PET-based and CSF-based amyloid measures and, where sample size permits, test effect modification by sex, APOE ε4 status, and cumulative vascular risk burden. Such standardized approaches can strengthen assessment of temporal ordering and help identify the most informative biomarkers for early preclinical detection and risk stratification.

Further mechanistic investigation is warranted to clarify the precise mechanisms linking Aβ to WM damage, including the roles of CAA, blood-brain barrier dysfunction, and neuroinflammation. Integrating advanced dMRI, including diffusion kurtosis imaging (DKI) and neurite orientation dispersion and density imaging (NODDI), with amyloid and tau PET and fluid biomarkers from CSF and plasma may provide complementary information about the underlying pathophysiology.

Ultimately, a deeper understanding of these interactive mechanisms is essential to inform disease-modifying strategies. The high prevalence of mixed pathology suggests that future therapeutic studies should test personalized approaches, targeting AD pathology, SVD, or both, depending on the individual's specific pathological profile. Their efficacy, safety, and timing require interventional evidence and cannot be inferred from the observational associations reviewed here.

This narrative review also has limitations. The available search description does not establish exhaustive retrieval, and no formal study-level risk-of-bias assessment or quantitative synthesis is reported. The cited evidence includes different clinical populations, imaging measures, cognitive outcomes, and follow-up intervals; selected symptomatic studies provide context rather than direct preclinical evidence. Several reports draw on the same underlying cohorts, including ADNI, and the dissertation and related abstract should not be treated as independent replication. These constraints limit conclusions about consistency, comparative predictive performance, and causal mechanisms.

## 5 Conclusion

In biomarker-characterized CU samples, WMHs and Aβ show independent and additive associations with cognition, with more variable evidence for statistical interaction and temporal coupling. Broader CU and mixed or symptomatic studies provide supportive evidence and should not define conclusions about preclinical AD. Regional WMH associations vary with anatomical definitions, population, and adjustment; posterior or periventricular patterns are not specific for amyloid, and frontal lesions are not uniformly vascular. Some longitudinal studies associate baseline Aβ with later WMH accrual, but neither this temporal ordering nor spatial overlap establishes causation. Tau associations should be distinguished by tissue compartment and imaging scale. Diffusion and disconnectome findings extend biological characterization beyond visible lesion volume; added individual prognostic value remains to be demonstrated. Longitudinal executive-function interactions from a dissertation and related abstract remain preliminary and non-independent. Formal interactions in other cohorts do not justify a universal synergistic model. WMH currently has a clearer role in trial eligibility and MRI safety assessment than as a validated treatment endpoint. Randomized and harmonized longitudinal evidence is needed before attributing chronic WMH change to plaque-clearing therapy or using these measures for preventive treatment selection.
